# Social relationships, emotion regulation, and emotional wellbeing in adolescence: associations in youth with and without a migratory background

**DOI:** 10.3389/fpsyg.2026.1847523

**Published:** 2026-10-06

**Authors:** Francesca Federico, Michela Mellone, Sergio Melogno, Margherita Orsolini

**Affiliations:** 1 Department of Developmental and Social Psychology, Sapienza University of Rome, Rome, Italy; 2 Faculty of Psychology, University of Niccolò Cusano, Rome, Italy

**Keywords:** adolescence, emotion regulation, emotional wellbeing, migratory background, school context, social relationships

## Abstract

Emotional wellbeing in adolescence develops within interconnected personal and relational systems, but the relative salience of family, peer, and school contexts may vary across sociocultural experiences. The present study examined associations among perceived functioning in school, family, and peer domains, everyday emotion-regulation difficulties, and affective emotional wellbeing in 79 third-year middle-school students in Rome (M age = 13.80 years, SD = 0.45; 45.6% female), including 42 adolescents without and 37 with a migratory background, defined as having at least one foreign-born parent. Participants completed self-report assessments of relational functioning across the three domains, emotion-regulation difficulties, and positive and negative affect. Two separately estimated observed-variable path models were used for exploratory purposes. Among adolescents with a migratory background, school (*β* = 0.44, *p* = 0.002) and family (*β* = 0.53, *p* < 0.001) indicators were independently associated with greater emotional wellbeing. No direct relational-context path to emotional wellbeing reached significance in the group without a migratory background. Emotion-regulation difficulties were not independently associated with emotional wellbeing in either group, and percentile bootstrap confidence intervals based on 5,000 resamples did not support indirect pathways through emotion regulation. The findings point to the possible importance of family and school as interconnected relational systems while providing no support for the hypothesized regulatory pathway in this sample. Given the modest subgroup sizes, cross-sectional design, single-item contextual indicators, and heterogeneous migratory-background category, the results should be considered exploratory and hypothesis-generating.

## Highlights

- Family and school relational-context indicators were independently associated with emotional wellbeing among adolescents with a migratory background.
- Emotion regulation difficulties were not independently associated with emotional wellbeing in the revised group-specific models.
- No indirect pathway through emotion regulation was supported by 5,000-resample bootstrap confidence intervals.
- Group-specific findings are exploratory and do not constitute formal evidence of between-group differences.

## Introduction

Adolescence is a period of substantial biological, cognitive, affective, and social reorganization, during which young people become increasingly responsive to the opportunities and demands of their social environments (Braams and Krabbendam, 2022; Galván, 2021). These changes unfold at a time when mental-health vulnerabilities become increasingly visible: recent World Health Organization estimates indicate that approximately one in seven 10- to 19-year-olds experiences a mental-health condition, while supportive family, peer, school, and community environments are recognized as important determinants of adolescent mental wellbeing (World Health Organization, 2025). Understanding development during this period therefore requires attention not only to distress and psychopathology, but also to the relational and individual processes that support positive emotional functioning.

Wellbeing is a multidimensional construct, and its terminology is not uniform. Subjective wellbeing is classically conceptualized as including both an affective component—the relative predominance of positive over negative affect—and a cognitive-evaluative component such as life satisfaction (Diener, 1984, 2009). Contemporary work on emotional wellbeing (EWB) similarly emphasizes affective experience while also recognizing broader reflective dimensions, including life satisfaction, meaning, and goal pursuit, all situated within culture, resources, life circumstances, and the life course (Park et al., 2023). Thus, subjective wellbeing and emotional wellbeing overlap but are not interchangeable. In the present conceptual framework, affective emotional wellbeing refers specifically to the experiential balance of positive and negative affect, while broader evaluative and eudaimonic components are treated as related but distinct aspects of wellbeing (Diener, 1984; Park et al., 2023).

Because adolescents’ affective experiences are embedded in everyday interactions, emotional wellbeing can be situated within a developmental systems perspective. Bronfenbrenner and Morris's (2006) bioecological model provides a specific account of this systems logic by describing development as emerging through reciprocal proximal processes between the person and nested social contexts over time. This perspective is especially relevant in adolescence, when increasing autonomy and social reorientation broaden young people’s participation across family, peer, and school settings and make development increasingly sensitive to contextual inputs (Galván, 2021; Veenstra and Laninga-Wijnen, 2022). Family, friendships, and school can therefore be understood as interconnected relational systems that provide different forms of support, feedback, belonging, and opportunities for participation rather than as isolated influences on wellbeing.

A systems perspective is particularly useful for understanding adolescents with a migratory background because migration-related development is heterogeneous and may reorganize both demands and resources across family, school, peer, community, and cultural contexts. A related, but conceptually distinct, multisystem resilience perspective focuses specifically on resilience: the capacity of dynamic systems to adapt successfully when significant challenges threaten function or development, through processes and resources distributed across multiple levels (Masten et al., 2021). In work focused on migrant youth, resilience has been conceptualized as a dynamic process in which young people mobilize and negotiate resources across intrapersonal, interpersonal, and institutional systems (Motti-Stefanidi, 2021; Wu and Ou, 2021). This perspective does not imply that migration itself constitutes uniform adversity, nor that all youth with a migratory background require resilience in the same way. Rather, it highlights why the availability and coordination of relational resources may be especially important when adolescents navigate developmental and sociocultural transitions.

Empirical research supports the relevance of these relational resources. Friendships become increasingly salient during adolescence and contribute to intimacy, validation, identity exploration, and emotional support (Furman and Buhrmester, 2009; Veenstra and Laninga-Wijnen, 2022). Family relationships remain important sources of security, continuity, and support even as autonomy increases (Branje, 2018; Morris et al., 2017). Schools provide sustained social environments in which adolescents interact with peers and adults, encounter expectations and norms, and experience inclusion, recognition, or exclusion (Eccles and Roeser, 2011). Among immigrant-background youth, family cohesion and school belonging have been identified as potentially protective relational resources (Shah et al., 2021), while recent longitudinal work shows that improvements in peer belonging and supportive school climate are associated with more positive emotional-health trajectories across immigrant, refugee, and non-immigrant early adolescents (Thomson et al., 2024). Other recent studies likewise underscore the relevance of parent-youth relationships and feelings about school for the social–emotional wellbeing of immigrant youth (Scheel et al., 2025). Whole-school approaches are consistent with this evidence in emphasizing that student mental health is shaped through relationships, inclusion, participation, school climate, and links between schools, families, and communities (Cefai et al., 2021).

Within these relational systems, emotion regulation (ER) represents a person-level process that may contribute to emotional wellbeing. ER refers to the processes through which people monitor, evaluate, and modify emotional responses in the service of goals (Gross, 2015). Adolescence is an important period for the refinement of regulatory capacities because cognitive development, expanding autonomy, and increasing social complexity create new regulatory demands and opportunities (Silvers, 2022). At the same time, ER is not exclusively intrapersonal: developmental accounts emphasize that regulatory capacities are shaped within relationships through modeling, emotional communication, support, and repeated experiences of co-regulation (Morris et al., 2017; Cole et al., 2019). Relational contexts may therefore be associated with emotional wellbeing both directly and through their associations with regulatory functioning, although the existence and direction of such pathways must be established empirically rather than assumed.

Taken together, developmental systems theory, research on relational resources in adolescence, multisystem perspectives on migrant youth, and work on emotion regulation converge on the need to examine emotional wellbeing across multiple levels of the adolescent’s ecology (Bronfenbrenner and Morris, 2006; Masten et al., 2021; Morris et al., 2017; Thomson et al., 2024). However, family, peer, and school contexts are often examined separately, making it difficult to determine whether each contributes unique information when the other relational contexts and individual regulatory difficulties are considered simultaneously. In addition, relatively little work has examined whether the same pattern of within-group associations is descriptively evident among adolescents with and without a migratory background. Addressing these gaps can help generate more precise hypotheses about how relational resources and regulatory processes jointly relate to adolescent affective wellbeing.

### Present study and hypotheses

The present study examined simultaneous associations among school, family, and peer relational-context indicators, emotion-regulation difficulties, and affective emotional wellbeing. Three hypotheses were specified: (H1) higher school, family, and peer indicators would be associated with greater emotional wellbeing; (H2) higher school, family, and peer indicators would be associated with fewer emotion-regulation difficulties; and (H3) greater emotion-regulation difficulties would be associated with lower emotional wellbeing. Statistical indirect pathways from each relational-context indicator to emotional wellbeing through emotion-regulation difficulties were examined as exploratory extensions of these hypotheses. Because migratory background was a central contextual feature of the study, we also explored the pattern of these associations separately among adolescents with and without a migratory background, without specifying directional hypotheses about between-group differences.

## Methods

### Participants and procedure

The study was approved by the Ethics and Research Committee of Sapienza University of Rome. Recruitment took place in two comprehensive schools in Rome between February and March 2024. The schools were purposively approached because both served substantial numbers of students with migratory backgrounds; one was located in central Rome and the other in a peripheral area. Recruitment targeted third-year classes of lower-secondary school. School staff coordinated access and scheduling with the research team; no random sampling of schools or classes was used. The available project records do not allow reconstruction of whether every eligible third-year class was approached, and this limitation of procedural documentation is acknowledged. Written informed consent was obtained from parents or legal guardians before participation.

Assessments were conducted during school hours in rooms made available for the project, on days and times coordinated with school staff. If a participant was tired or did not wish to continue, the session could be interrupted and rescheduled. A total of 79 third-year students were included in the final analytic sample (36 females, 43 males; *M* age = 13.80 years, SD = 0.45, range = 13–14 years).

Migratory background was reconstructed directly from parental country-of-birth fields. Adolescents were classified as having a migratory background when at least one parent was born outside Italy, yielding 42 adolescents without and 37 with a migratory background. Within the latter group, 27 had two foreign-born parents and 10 had one foreign-born parent; 10 adolescents were themselves born outside Italy and 27 were born in Italy. The grouping therefore combines heterogeneous migration histories and should not be interpreted as a homogeneous sociocultural category.

### Measures

Raven’s Standard Progressive Matrices (SPM). The SPM was administered as a nonverbal measure of general reasoning (Raven et al., 2000). It was originally included in the project as a screening measure to identify marked cognitive difficulties that might interfere with comprehension of the study procedures. In the archived dataset, standard-score information was available for 51 participants: 50 scores were ≥80 and one score was 78, while 28 scores were unavailable. Because the archived Raven data were incomplete, SPM performance was not used as an exclusion criterion for the present analytic sample. A sensitivity analysis excluding the one participant with an observed score below 80 (*N* = 78) yielded the same substantive pattern of findings.

Behavior Rating Inventory of Executive Function—Second Edition, Self-Report (BRIEF-2 SR). Emotion regulation difficulties were indexed by the Emotional Regulation Index (ERI) T-score of the BRIEF-2 Self-Report (Gioia et al., 2016). The ERI combines the Emotional Control and Shift scales; higher T-scores indicate greater everyday regulation difficulties. Item-level BRIEF-2 responses were not retained in the archived analytic dataset, so a sample-specific item-level reliability coefficient for the ERI could not be recomputed. We report this limitation explicitly rather than inferring reliability from the composite T-score.

Definitional Positive and Negative Affect Schedule for Children (d-PANAS-C). Affective emotional wellbeing was assessed with the 10-item d-PANAS-C (Smees et al., 2020), comprising five positive-affect and five negative-affect items rated on a five-point response scale. Positive Affect (PA) and Negative Affect (NA) were computed as item means; the emotional wellbeing index was PA-NA, with higher scores indicating a more positive affective balance. In the analytic sample, Cronbach’s *α* was 0.78 for PA and 0.73 for NA. When negative-affect items were reverse scored, internal consistency across the 10 affect-balance items was *α* = 0.82.

Very Short Wellbeing Questionnaire for Children (VSWQ-C). The VSWQ-C includes four brief domain items concerning how well children feel in class, at home, with friends, and with their body (Smees et al., 2020). The present study used the class, home, and friends items separately as single-item indicators of the school, family, and peer relational contexts; the body item was not included in the model. Scores ranged from 1 (never) to 5 (always), with higher values indicating more positive perceived functioning in that domain. Because each context is represented by a single item, conventional internal-consistency coefficients are not applicable and measurement error cannot be estimated. Accordingly, we use the terms school, family, and peer domain indicators rather than treating these items as multi-item measures of broader constructs such as school connectedness.

Sociocultural questionnaire. An *ad hoc* questionnaire recorded adolescents’ and parents’ country of birth, family composition, languages used at home, and related sociocultural information. These data were used descriptively and to reconstruct migratory-background classification directly from parental country of birth.

### Data analysis

The original submission referred to a multi-group path analysis. The revised analysis is more accurately described as two separately estimated group-specific observed-variable path models. In each group, school, family, and peer domain indicators were entered simultaneously as predictors of ERI and of emotional wellbeing, and ERI was entered as an additional predictor of emotional wellbeing. The three exogenous domain indicators were allowed to covary. This produces seven focal regression paths per group. Because the observed-variable specification is just-identified/saturated, global fit indices are not informative and are therefore not interpreted. The group-specific models were used descriptively and for hypothesis generation; no formal test of between-group coefficient differences was conducted.

All analyses were recomputed from the source dataset using Python 3.13.5 with NumPy 2.3.5 and SciPy 1.17.0. All 79 participants had complete data on the focal model variables; analyses therefore used complete cases, no imputation was required, and no participant was excluded because of missing focal data. Missing Raven scores did not affect model estimation because Raven performance was not included as a model variable or analytic exclusion criterion. Before model estimation, the focal variables were standardized within each migratory-background group; consequently, the reported *β* coefficients, standard errors, and confidence intervals are on the within-group standardized scale. For observed continuous variables in a saturated path model, the regression-path estimates are equivalent to ordinary least-squares regression coefficients. We report standardized path coefficients (*β*), standard errors, 95% confidence intervals, and two-sided *p* values for direct paths. Statistical significance for direct paths was defined at *α* = 0.05; values between 0.05 and 0.10 were treated as non-significant and were not labeled as trends. The model *R^2^* is reported for ERI and emotional wellbeing in each group.

Indirect associations were calculated as the product of the relevant standardized a path (domain indicator → ERI) and b path (ERI → emotional wellbeing). Their uncertainty was evaluated using 5,000 nonparametric bootstrap resamples and percentile 95% confidence intervals. For these indirect pathways, the bootstrap confidence interval—not a separate parametric *p* value—was the inferential criterion: an interval including zero was interpreted as not supporting the indirect association. This approach avoids the inconsistency in the submitted manuscript, in which a parametric *p* value and a bootstrap confidence interval led to different conclusions. Given the cross-sectional design, all quantities are described as associations or statistical pathways rather than causal effects or mediation mechanisms. No formal test of between-group coefficient differences was conducted.

## Results

### Descriptive statistics and bivariate associations

Table 1 presents descriptive statistics by migratory-background group and bivariate Pearson correlations for the full sample. Emotional wellbeing showed positive zero-order correlations with the school (*r* = 0.52), family (*r* = 0.56), and peer (*r* = 0.30) domain indicators. ERI was negatively correlated with family (*r* = −0.25) and peer (*r* = −0.27) indicators and showed a small negative zero-order correlation with emotional wellbeing (*r* = −0.15).

**Table 1:** Descriptive statistics by group and bivariate correlations in the full sample.

Variable	Total *M* (SD)	No migratory background *M* (SD)	Migratory background *M* (SD)	1	2	3	4
1. School domain indicator	2.97 (0.99)	3.17 (0.66)	2.76 (1.23)	–			
2. Family domain indicator	3.28 (1.00)	3.55 (0.74)	2.97 (1.17)	0.40	–		
3. Peer domain indicator	3.37 (0.77)	3.55 (0.71)	3.16 (0.80)	0.25	0.51	–	
4. Emotion regulation difficulties (ERI)	60.95 (12.63)	58.83 (13.39)	63.35 (11.42)	−0.07	−0.25	−0.27	–
5. Emotional wellbeing (PA-NA)	1.57 (1.42)	1.92 (1.16)	1.17 (1.60)	0.52	0.56	0.30	−0.15

*N* = 79 (42 without and 37 with a migratory background). Correlations are Pearson correlations in the full sample. ERI, Emotional Regulation Index; higher ERI scores indicate greater difficulties. PA, Positive Affect; NA, Negative Affect.

### Group-specific standardized path coefficients

Among adolescents without a migratory background (*n* = 42), none of the adjusted direct paths from the three domain indicators to emotional wellbeing reached *p* < 0.05. The peer domain indicator was not significantly associated with ERI (*β* = −0.30, *p* = 0.072), and neither the school nor family indicator was significantly associated with ERI. ERI was not independently associated with emotional wellbeing (*β* = −0.10, *p* = 0.556). The model explained 21.3% of the variance in ERI and 18.5% of the variance in emotional wellbeing.

Among adolescents with a migratory background (*n* = 37), the school domain indicator was independently associated with greater emotional wellbeing (*β* = 0.44, *p* = 0.002), as was the family domain indicator (*β* = 0.53, *p* < 0.001). The peer indicator was not independently associated with emotional wellbeing. None of the three domain indicators was significantly associated with ERI, and ERI was not independently associated with emotional wellbeing (*β* = 0.04, *p* = 0.736). The model explained 1.8% of the variance in ERI and 55.2% of the variance in emotional wellbeing. These results describe associations within each subgroup; because no formal coefficient-difference tests were conducted, they do not demonstrate statistically significant differences between groups (Table 2).

**Table 2:** Full decomposition of direct and indirect statistical pathways by migratory background.

Path	Group	*β*	SE	95% CI	*p*
School → ERI	No MB	0.002	0.159	[−0.320, 0.323]	0.992
Family → ERI	No MB	−0.244	0.170	[−0.589, 0.100]	0.159
Peer → ERI	No MB	−0.299	0.161	[−0.625, 0.028]	0.072
School → Emotional wellbeing	No MB	0.117	0.164	[−0.214, 0.449]	0.479
Family → Emotional wellbeing	No MB	0.244	0.180	[−0.121, 0.609]	0.184
Peer → Emotional wellbeing	No MB	0.118	0.174	[−0.234, 0.470]	0.500
ERI → Emotional wellbeing	No MB	−0.099	0.167	[−0.438, 0.239]	0.556
School → ERI → Emotional wellbeing	No MB	−0.000	0.047	[−0.110, 0.098]	–
Family → ERI → Emotional wellbeing	No MB	0.024	0.058	[−0.067, 0.173]	–
Peer → ERI → Emotional wellbeing	No MB	0.030	0.066	[−0.091, 0.190]	–
School → ERI	MB	0.114	0.184	[−0.259, 0.488]	0.538
Family → ERI	MB	−0.124	0.210	[−0.552, 0.303]	0.558
Peer → ERI	MB	0.009	0.201	[−0.399, 0.417]	0.965
School → Emotional wellbeing	MB	0.436	0.127	[0.178, 0.694]	0.002
Family → Emotional wellbeing	MB	0.525	0.145	[0.230, 0.820]	<0.001
Peer → Emotional wellbeing	MB	−0.125	0.138	[−0.405, 0.156]	0.372
ERI → Emotional wellbeing	MB	0.041	0.119	[−0.202, 0.284]	0.736
School → ERI → Emotional wellbeing	MB	0.005	0.025	[−0.043, 0.064]	–
Family → ERI → Emotional wellbeing	MB	−0.005	0.027	[−0.073, 0.043]	–
Peer → ERI → Emotional wellbeing	MB	0.000	0.029	[−0.054, 0.066]	–

No MB = without a migratory background; MB = with a migratory background. Direct-path confidence intervals and *p* values are based on the regression model. For indirect pathways, SE is the bootstrap standard error and the 95% CI is the percentile interval based on 5,000 resamples; no separate parametric *p* value was used because the bootstrap interval was the inferential criterion. Higher ERI scores indicate greater emotion regulation difficulties.

### Indirect statistical pathways

None of the 5,000-resample percentile bootstrap intervals supported an indirect association through ERI. In adolescents without a migratory background, the standardized indirect estimates were approximately 0.000 for school (95% bootstrap CI [−0.110, 0.098]), 0.024 for family [−0.067, 0.173], and 0.030 for peers [−0.091, 0.190]. In adolescents with a migratory background, corresponding estimates were 0.005 for school [−0.043, 0.064], −0.005 for family [−0.073, 0.043], and approximately 0.000 for peers [−0.054, 0.066]. Thus, the indirect school → ERI → emotional wellbeing pathway reported in the submitted version was not reproduced when the source dataset and outcome scoring were rechecked and the analysis was rerun with a single, consistent bootstrap inferential criterion (Figures 1 and 2).

**Figure 1:**
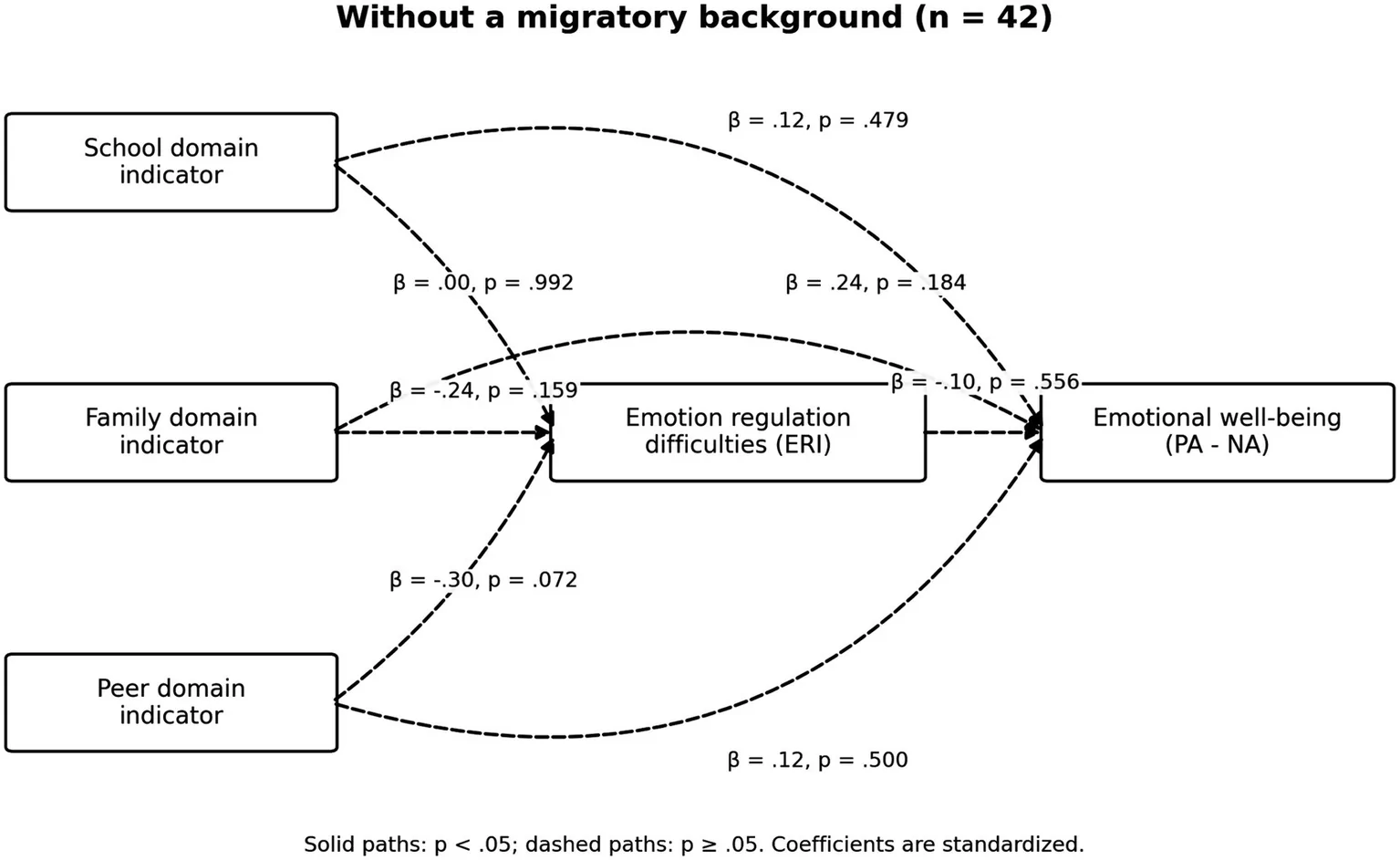
Group-specific path model for adolescents without a migratory background. Standardized coefficients are shown. Solid paths indicate *p* < 0.05; dashed paths indicate *p* ≥ 0.05. The model was estimated separately and does not constitute a formal between-group test.

**Figure 2:**
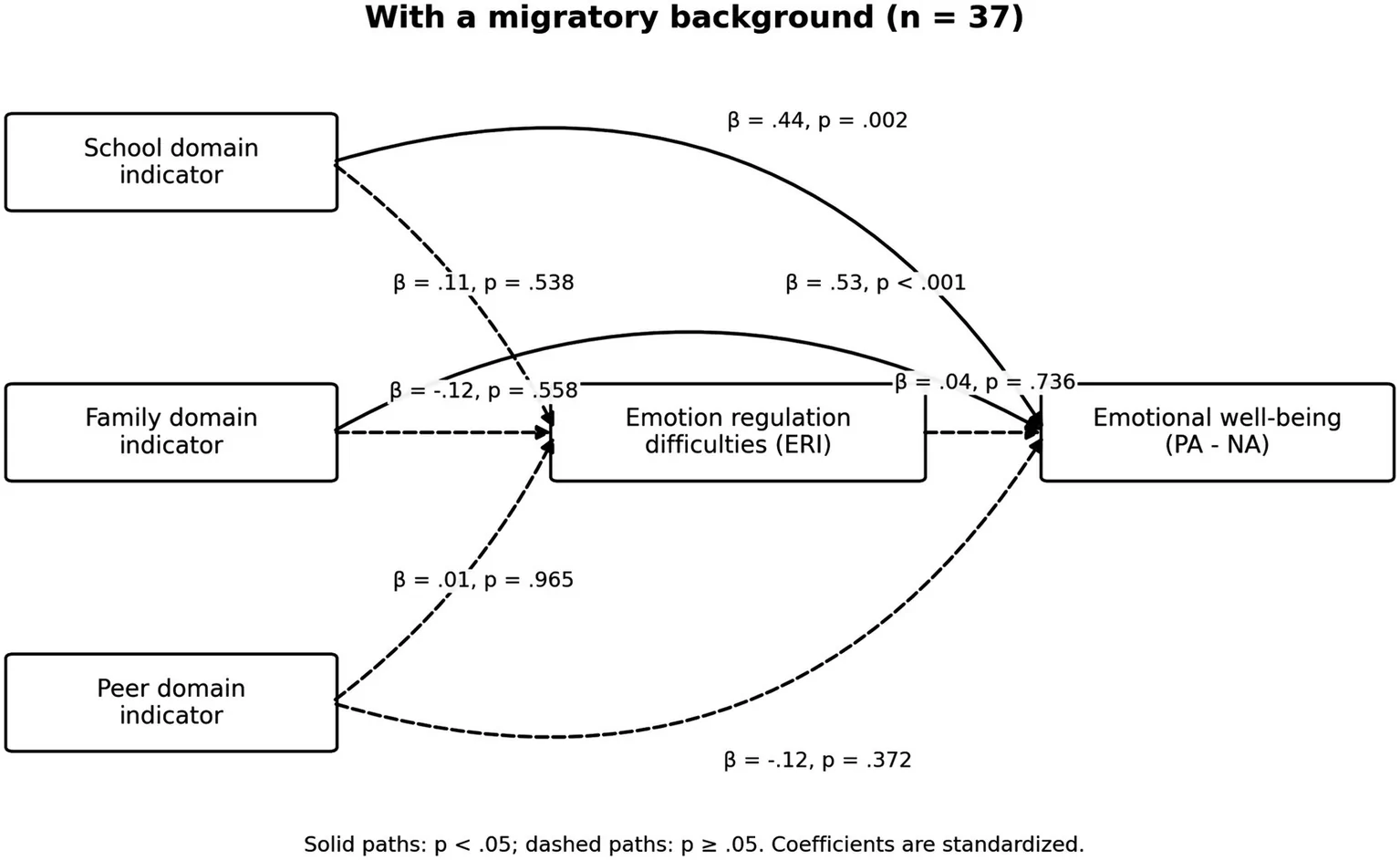
Group-specific path model for adolescents with a migratory background. Standardized coefficients are shown. Solid paths indicate *p* < 0.05; dashed paths indicate *p* ≥ 0.05. The model was estimated separately and does not constitute a formal between-group test.

## Discussion

The revised analyses were interpreted in relation to the three hypotheses specified *a priori*. H1, which predicted positive associations between the school, family, and peer domain indicators and emotional wellbeing, received partial support. Within the subgroup of adolescents with a migratory background, both the school and family indicators were independently associated with greater affective emotional wellbeing after the other relational domains and ERI were included in the model. The peer indicator was not independently associated with emotional wellbeing in this subgroup, and none of the three relational indicators reached significance in the subgroup without a migratory background. These patterns are descriptive within-group findings rather than evidence that the corresponding coefficients differ significantly between groups.

The school and family findings in the migratory-background subgroup are consistent with recent work emphasizing relational resources across multiple contexts of immigrant adolescents’ lives. Longitudinal evidence indicates that improvements in supportive school climate and peer belonging are associated with more positive emotional-health trajectories among immigrant and refugee as well as non-immigrant early adolescents (Thomson et al., 2024). Research focused specifically on immigrant youth has also identified family cohesion and school belonging as resources associated with more favorable socioemotional outcomes under conditions of bias-based bullying (Shah et al., 2021), and recent work has linked student-parent relationship quality and feelings about school to emotional distress and social wellbeing among bullied immigrant youth (Scheel et al., 2025). From a bioecological perspective, the relevance of family and school is plausible because both are proximal settings in which repeated interactions can provide support, recognition, continuity, and opportunities for participation (Bronfenbrenner and Morris, 2006). Whole-school models similarly emphasize that student wellbeing is influenced not only by individual skills but also by relationships, inclusion, school climate, and school-family-community links (Cefai et al., 2021). Our single-item school and family indicators cannot identify which of these processes account for the observed associations, but the findings support examining them directly in future research.

The absence of an adjusted peer association does not imply that peer relationships are unimportant. In the full sample, the peer indicator showed a positive zero-order correlation with emotional wellbeing, and extensive adolescent research identifies friendships and peer belonging as important sources of support and adjustment (Veenstra and Laninga-Wijnen, 2022; Thomson et al., 2024). The loss of significance in the multivariable path models may reflect overlap among relational contexts: the peer indicator correlated with family and school indicators, so its unique coefficient represents only the variance not shared with those other settings. With small subgroups and single-item indicators, the present study has limited capacity to disentangle these overlapping relational contributions. The lack of significant relational paths in the group without a migratory background should likewise not be interpreted as evidence that family, peers, or school matter less for these adolescents; formal interaction or equality tests in larger samples are required before drawing such conclusions.

H2, which predicted that more positive school, family, and peer indicators would be associated with fewer emotion-regulation difficulties, was not supported at the prespecified α = 0.05 level. None of the three relational indicators showed a significant adjusted association with ERI in either subgroup. This null finding does not contradict the broader proposition that emotion regulation develops in relational contexts, but it suggests that the specific cross-sectional associations tested here were not detectable with the present measures and sample. Developmental models describe emotion regulation as socially embedded through modeling, emotional communication, and co-regulation (Morris et al., 2017), while adolescent regulation is also highly sensitive to context and to the particular strategy or regulatory demand being assessed (Silvers, 2022). The BRIEF-2 ERI is a broad everyday index combining emotional control and shifting, whereas the relational predictors were single domain items. This difference in construct breadth may have reduced correspondence between predictors and outcome.

H3, which predicted that greater emotion-regulation difficulties would be associated with lower emotional wellbeing, was also not supported. ERI was not independently associated with affective emotional wellbeing in either subgroup after the relational indicators were included, and none of the exploratory indirect pathways through ERI was supported by the 5,000-resample bootstrap intervals. Emotion regulation remains theoretically and empirically relevant to adolescent adjustment (Gross, 2015; Silvers, 2022), but the literature also shows substantial heterogeneity in how regulation is operationalized and measured across adolescent studies (Eadeh et al., 2021). In the present study, ERI indexes broad self-perceived regulatory difficulties, whereas emotional wellbeing reflects short-term affective balance. The absence of an association may therefore reflect measurement alignment, limited statistical power, shared variance with relational indicators, or a genuinely weak relation between these specific operationalizations. Longitudinal work using strategy-specific, contextual, or intensive repeated measures of regulation would provide a stronger test of the proposed developmental pathway.

The findings can be situated within a multisystem resilience perspective, but only with conceptual caution. Resilience is not synonymous with adjustment in general; it concerns successful adaptation in the context of significant challenge and depends on capacities and resources distributed across interacting systems (Masten et al., 2021). Multisystem approaches to migrant youth similarly emphasize that young people may mobilize resources across interpersonal and institutional settings and that the relevance of particular resources depends on the challenges and contexts involved (Motti-Stefanidi, 2021; Wu and Ou, 2021). Because the present study did not assess exposure to adversity or resilience processes directly, it does not demonstrate resilience. Rather, the independent family and school associations observed within the migratory-background subgroup are compatible with the broader proposition that relational resources can be distributed across multiple systems and should be studied jointly rather than in isolation.

The conceptualization of emotional wellbeing also shapes interpretation. Park et al. (2023) describe EWB as a broad construct that includes experiential and reflective aspects of positive functioning situated in context. The present outcome captures the affective component of this broader domain. Accordingly, the observed associations should not be generalized automatically to life satisfaction, meaning, purpose, or other evaluative and eudaimonic dimensions. Future studies using multidimensional wellbeing measures could test whether family, peer, school, and regulatory processes show different patterns across affective and reflective components.

### Strengths, limitations, and future directions

Several features strengthen the contribution of the study. First, family, peer, and school relational contexts were considered simultaneously rather than in separate models, allowing their adjusted associations with emotional wellbeing to be examined within the same conceptual framework. Second, the study places individual emotion-regulation difficulties alongside relational contexts, consistent with a multilevel developmental perspective. Third, migratory background was treated as a heterogeneous contextual characteristic rather than as an inherent deficit, and the revised analyses explicitly distinguish descriptive within-group patterns from formal evidence of between-group differences. Finally, the reanalysis reports the full set of direct and indirect pathways with a consistent bootstrap criterion, increasing methodological transparency.

These strengths should be considered alongside important limitations. The sample is small for group-specific path modeling: with 42 and 37 adolescents, parameter estimates are imprecise, no *a priori* power analysis was available, and some coefficients may be sample-dependent. The school, family, and peer predictors are single-item domain indicators, which limits construct coverage, increases measurement error, and precludes internal-consistency estimation. Item-level BRIEF-2 data were unavailable in the archived analytic dataset, preventing recomputation of sample-specific ERI reliability. All focal variables were self-reported, raising the possibility of shared-method variance. The cross-sectional design establishes contemporaneous associations only and cannot support temporal or causal mediation. Migratory background was operationalized broadly as at least one foreign-born parent, combining first-generation, second-generation, and mixed-parentage experiences; the sample is too small for meaningful subgroup analyses. Raven data were incomplete and could not serve as a uniform cognitive-screening criterion, and the archival procedural documentation does not allow a complete reconstruction of class-level recruitment rates.

Future research should recruit substantially larger and more diverse samples, preregister power calculations and analytic plans, and use multi-item measures that distinguish specific family, peer, and school processes. Direct assessment of school belonging and climate, parent-adolescent relationship quality, discrimination, acculturative experiences, and other migration-related resources and stressors would allow the proposed systems interpretation to be tested rather than inferred. Longitudinal or intensive repeated-measures designs would be particularly valuable for examining temporal relations among relational contexts, emotion regulation, and wellbeing. Multidimensional measures of wellbeing could determine whether contextual associations differ across affective, evaluative, and eudaimonic components. Finally, formal moderation or multi-group equality tests should be used when the research question concerns differences in path strength across sociocultural groups.

## Conclusion

Adolescent affective wellbeing is embedded in multiple relational systems. In this exploratory study, H1 received partial support: family and school domain indicators showed independent positive associations with emotional wellbeing within the subgroup of adolescents with a migratory background, whereas H2 and H3 were not supported and the exploratory indirect pathways through emotion-regulation difficulties were not evident. These findings do not establish between-group differences, causal mechanisms, or resilience processes. They instead support a context-sensitive research agenda in which relational resources, individual regulatory processes, migratory experiences, and multiple dimensions of wellbeing are measured with greater precision and examined over time.

## Data Availability

The raw data supporting the conclusions of this article will be made available by the authors, without undue reservation.
